# Add-on topiramate reduces weight in overweight patients with affective disorders: a clinical case series

**DOI:** 10.1186/1471-244X-5-19

**Published:** 2005-04-07

**Authors:** George Kirov, John Tredget

**Affiliations:** 1 Department of Psychological Medicine, School of Medicine, Cardiff University, Heath Park, Cardiff, CF14 4XN, UK

## Abstract

**Background:**

The weight-gain caused by many psychotropic drugs is a major cause for poor compliance with such medications and could also increase cardio-vascular morbidity among psychiatric patients. Recent reports have shown that the anticonvulsant topiramate causes weight loss in various patient groups. The drug has also shown effectiveness in open trials as a mood stabilizer in patients with affective disorders, but not in controlled trials in the acute treatment of mania. We used topiramate to treat 12 patients with affective disorders who had a body-mass index >30 kg/m^2^.

**Methods:**

Topiramate was prescribed as part of our routine clinical practice, as an add-on medication, or as a replacement of a mood stabilizer. Patients' weight was recorded in 1 to 2 monthly intervals. Patients were followed up for between 6 and 12 months. The final dose of topiramate varied from 200 to 600 mg/day.

**Results:**

Topiramate was effective in reducing the weight in 10 out of the 12 patients. At six months the 12 patients had lost a mean of 7.75 kg (SD = 6.9 kg, p < 0.001) and at 12 months 9 patients had lost a mean of 9.61 kg (SD = 6.7 kg, p = 0.003). Three patients stopped the treatment: one due to side effects, one due to possible side effects, and one suffered a manic relapse and showed no sustained weight loss. There were no other clear changes in the course of illness of the patients.

**Conclusion:**

The evidence of a strong weight-reducing potential of topiramate is indisputable and clinically significant. Topiramate could be considered in the treatment of bipolar patients who are overweight, or whose concerns about weight gain compromise their compliance with long-term prophylactic medication. So far there is no evidence that topiramate has anti-manic effect and it should not be used as monotherapy.

## Background

Psychiatric patients who receive long-term mood-stabilizing agents can gain excess body weight [1-3]. Weight gain is induced by the majority of antipsychotics [1,4], lithium [2,5,6], antidepressants [7] and valproate [5,8,9]. This could be a major problem with compliance and is one of the main reasons for discontinuation of treatment [1,10]. Increased body weight is also a major health hazard. Mortality rates for both men and women show a strong association with the body mass index (BMI) [11]. The risk starts to increase when the BMI is >25 kg/m^2 ^and doubles for BMI values of >40 kg/m^2^, where the correlation curve becomes progressively steeper [11]. It has been estimated that obesity causes 300,000 deaths per year in the USA alone [12].

Topiramate is a relatively new antiepileptic drug. Its effects include sodium-channel-blocking activity, enhancement of cerebral GABA concentrations and antagonism of AMPA/kainate receptors, which leads to a decreased glutamate-mediated excitation [13]. It has been shown to cause weight loss in a variety of treatment populations. In an add-on uncontrolled study on 34 epileptic patients there was a 5.9 kg weight loss at 1 year, while among the obese patients in that trial (BMI> = 30 kg/m^2^) the weight loss reached 10.9 kg [14]. The weight reducing potential of topiramate has been used to treat obesity in subjects with no concomitant psychiatric disorder or epilepsy. Bray et al, [15] treated 385 subjects in a randomised, double-blind, placebo-controlled study and reported weight losses of 4.8%–6.3% of body weight for increasing doses of topiramate treatment. Topiramate was also effective in the treatment of binge-eating disorder. In an open-label study of 13 patients topiramate resulted in 11.8 kg mean weight loss over a period of 3–30 months [16]. McElroy et al, [17] treated 61 outpatients affected with binge-eating associated with obesity for 14 weeks in a placebo-controlled trial. Mean weight loss was 5.9 kg and the binge-eating frequency was also reduced. The beneficial effects on weight and binge-eating were sustained during a 42-weeks open continuation of that trial [18], but a number of patients dropped out from the study. Another double-blind study on 69 patients with bulimia nervosa reported improvements in self-esteem and anxiety, in addition to the behavioural dimensions of bulimia [19].

Several studies have investigated the use of topiramate in bipolar disorders. Marcotte [20] evaluated 58 psychiatric patients, mostly with bipolar affective disorder. They were treated with add-on topiramate for a mean of 16 weeks. 62% were rated improved (weight changes were not reported). McElroy et al, [21] gave topiramate to 56 outpatients, either manic, depressed, or euthymic. The manic patients were reported improved, while the depressed (N = 11) did not change. Significant decreases in weight and BMI were recorded at every follow-up point, reaching 6.0 kg after 1 year. Gupta et al [22] performed a retrospective chart review of 5 patients with bipolar or schizoaffective disorder and reported a mean weight loss of 10 kg. Vieta et al, [23] treated 25 patients with treatment-resistant bipolar spectrum disorders in an open study and reported significant improvements in rating scales for depression and mania. Over 50% of all patients were considered to be responding to topiramate. Ten patients in that study experienced weight loss. Guille and Sachs, [24] treated 14 patients with refractory bipolar illness for an average of 22.4 weeks. Nine of them (64%) improved. Four patients with BMI>28 kg/m^2 ^experienced mean weight loss of 13.5 kg. In an open study topiramate was given together with Risperidone to 58 bipolar patients during a manic episode, of whom 41 completed the 12 months follow-up [25]. A significant improvement in rating scales for mania was recorded and relapse rates were lower compared to the preceding year. The mean weight of patients decreased by 1.1 kg by the end of the study. The same team examined the effect of the co-administration of topiramate and olanzapine over a 12 months interval [26]. Thirteen patients completed 12 months of treatment and their mania and depression ratings improved significantly. At the endpoint they showed 0.5 kg of weight loss, an impressive result given the known problems of weight gain associated with olanzapine [4]. A study using retrospective chart review on patients given topiramate showed a mean loss of 1.2 kg [27]. Topiramate was also used to treat acute manic episodes. In one study 18 patients with manic, mixed, or rapid-cycling episodes, resistant to current mood-stabilizers were given topiramate [28]. There were 12 patients who were judged responders after 5 weeks, and they had a mean weight loss of 4.3 kg. In a study conducted by Grunze et al, [29] 11 patients were given add-on topiramate, of whom 7 showed good improvement. In a study reported by Bozikas et al, [30], 14 manic patients received topiramate either as monotherapy, or in combination with antipsychotics for 4 weeks. Response rate was 61.5% and all patients tolerated topiramate well. Both weight loss and weight gain were reported in that study. Calabrese et al, [31] treated 10 patients hospitalised with mania with topiramate monotherapy for 4 weeks and 8 patients were described as responding. In a retrospective chart review of 76 patients, 13% showed a moderate to marked improvement on the Clinical Global Impression scale (CGI) [32]. There was a weight loss in 50% of patients in that sample, with a mean weight loss of 6.5 kg. A better response and higher weight loss correlated with higher doses of medication. Topiramate has been used in the treatment of the depressive phase of bipolar disorder. McIntyre et al, [33] compared bupropion and topiramate in 36 depressed BP outpatients in an open trial lasting 8 weeks. There was a similarly good improvement in both groups, with a 56% response rate on the Hamilton Rating Scale for Depression in the topiramate group (N = 18). The weight loss in this study was 5.8 kg in the topiramate group and 6 patients withdrew due to side effects. Lykouras and Hatzimanolis, [34] gave topiramate to 56 patients who had suffered relapses of bipolar disorder in the previous 12 months as an add-on treatment. There was a significant reduction of new manic and depressive episodes in the 50 patients who completed 12 months treatment.

All these open studies should be considered in the context of four large unpublished placebo-controlled trials of topiramate for the treatment of acute mania, which produced negative results (cited in Yatham [35]). They indicate clearly that monotherapy with topiramate has no acute anti-manic property. In contrast, almost all of the above open trials used topiramate as an add-on therapy.

Many patients in our Affective Disorders Clinic were concerned with excessive weight gain which they blamed at least partly on their treatments. As weight loss was a listed side effect of topiramate and several small studies had already reported on its use in bipolar affective disorders, we decided to use it as an add-on mood stabilizer in patients who had weight problems. We reasoned that topiramate could also have mood-stabilizing properties, like several other anticonvulsants, a finding already reported in open trials. We also felt that this off-licence use of topiramate was justified, as this intervention enabled patients to comply better with the rest of their medication, thus reducing potential relapses of illness, and that the weight loss could improve their risk for cardio-vascular disorders. We were not aware of the results from the controlled trials until all the patients described here were already taking topiramate.

### Patients

Patients in our mood disorders clinic are seen in regular intervals for long-term maintenance of their illness (some have attended for over 7 years now). As part of the service that we provide, patients have regular blood tests, reviews of medication and checks of weight, usually at intervals of 3 or 4 months. Topiramate was given to some obese patients as part of our normal clinical practice, rather than as an experimental intervention (therefore we sought no Ethics committee approval). We decided to publish our results only after the patients had taken topiramate for a considerable time, because we felt that we should share our experience with other health professionals, as the results looked so clear. The only difference to treatment as usual was that we checked the weight of these patients on a more regular basis than we would have normally done. All 12 patients included in this report had a pre-topiramate BMI of >30 kg/m^2^, i.e. they were all obese according to the National Heart, Lung and Blood Institute guidelines [36]. According to the British National Formulary guidelines [37], an anti-obesity drug should be considered only for those with a BMI of 30 or greater (page 207), and we felt that this recommendation should be followed in the case of Topiramate, even if this drug is not licenced for this use. The mean BMI of patients at the start was 37.95 kg/m^2^, SD = 5.66 and the mean weight was 104.38 kg, SD = 21.46 kg. The patients had the following diagnoses: Bipolar Affective Disorder type I (BPI) = 5, BPII = 4 and Unipolar Depression (UD) = 3. Four of them were rapid cyclers. Two of the three unipolar patients had co-morbid epilepsy and the third one reached a BMI of nearly 45 (extreme obesity). We felt that it was justified to give her this medication on general health grounds, although benefits from topiramate on unipolar depression had not been demonstrated before. Our clinic treats chronic and treatment resistant patients, and 9 of the 12 patients could be described as treatment resistant or incomplete responders, who had either frequent relapses of mood disorder (more than 2 per year), or remained chronically depressed (i.e. had not reached criteria for remission for a period of at least several weeks over the previous two years) despite adequate treatment. One more overweight patient was prescribed topiramate. He did not like its effect and stopped treatment after one month. He is therefore not included in any analysis.

### Concomitant medication

The nine treatment resistant/incomplete responder patients took combinations of medications: all of them took antidepressants, seven took antipsychotics, five took lithium and five took anticonvulsants (Table 1). One other patient was in a full remission on lithium monotherapy and one had recently been discharged from hospital after a manic relapse, having been relatively stable for several years prior to that. In four cases an anticonvulsant was exchanged with topiramate, in the remaining cases topiramate was given as an add-on treatment. No one received monotherapy of topiramate.

**Table 1:** Characteristics of the patients and changes of their weight during the study. Both individual and mean weight changes are presented. p-values are based on paired samples tests for the patients who have completed the corresponding follow-up interval. The dose of topiramate is the highest dose reached. Under "Concomitant medication" the drugs that are undelined were exchanged with topiramate.

Patient N	Gender	Age	DSM-IV	Concomitant medication	Topiramate dose (mg)	Start		Weight change from start (months)			
						
						BMI (kg/m^2^)	weight (kg)	3 months	6 months	9 months	12 months
1	f	49	UD	Nortryptiline, carbamazepine	600	42.4	110	-8	-10	-11	-12.5
2	f	48	BPI	Risperidone, lamotrigine, lofepramine	400	37.2	87	-9.5	-14	-12	-8
3	f	49	BPI	Quetiapine, mirtazapine, lithium, venlafaxine	200	42.3	99	-1	-5	-11	-14
4	m	50	BPII	Lithium, paroxetine	300	47.7	146	-10.5	-21	-26	-12.5
5	f	28	BPI	Lithium	200	31.5	79	-6	-10.5		
6	m	31	BPII	Lithium, reboxetine, trazodone, valproate	300	40.5	120	-6	-7.5	-6.5	-8
7	m	40	BPI	Olanzapine, fluoxetine	400	30.3	102.5	-1	+5	-1.5	-5.5
8	f	41	BPII	Fluphenazine, carbamazepine, paroxetine	250	34.7	90	-7	-14	-18	-22.5
9	f	70	UD	Mirtazapine, lithium, amisulpride, venlafaxine	200	44.4	116.5	-3	-4	-4	-4
10	f	54	BPII	Lithium, clomipramine, flupenthixol, trazodone	250	34.7	80	-1.5	-2.5	-2	+0.5
11	f	48	UD	Phenelzine, amisulpride, valproate	250	31.0	88.5	-3.5	-8.5		
12	m	33	BPI	Lithium, carbamazepine	200	38.7	134	-2.5	-1		
Mean reduction (kg)								4.96	7.75	10.2	9.61
SD								3.33	6.9	7.98	6.69
p-value								0.001	0.003	0.005	0.003

### Topiramate dosage

Topiramate dose was increased in steps of 25–50 mg every one to two weeks. After some of the first patients complained of paraesthesia and headaches, we routinely started making the increases only at 2-weekly intervals. The final dose of topiramate ranged between 200 and 600 mg, with a mean of 296 mg. The dose was increased until we could see a clear effect on the weight, or the patient reported some side effects. The patient whose topiramate was increased to 600 mg/day suffers with poorly controlled epilepsy and we increased the dose in order to achieve a better seizures control, rather than weight loss.

### Statistical analysis

The level of statistical significance for weight loss at each time-point during the follow-up, compared to baseline, was determined with a paired-samples t-test. This test assumes that comparisons at the four time points are independent, whreas the observations within individuals are, of course correlated. Therefore the test for statistical significance is slightly anti-conservative.

## Results

### Weight changes

**Figure 1:**
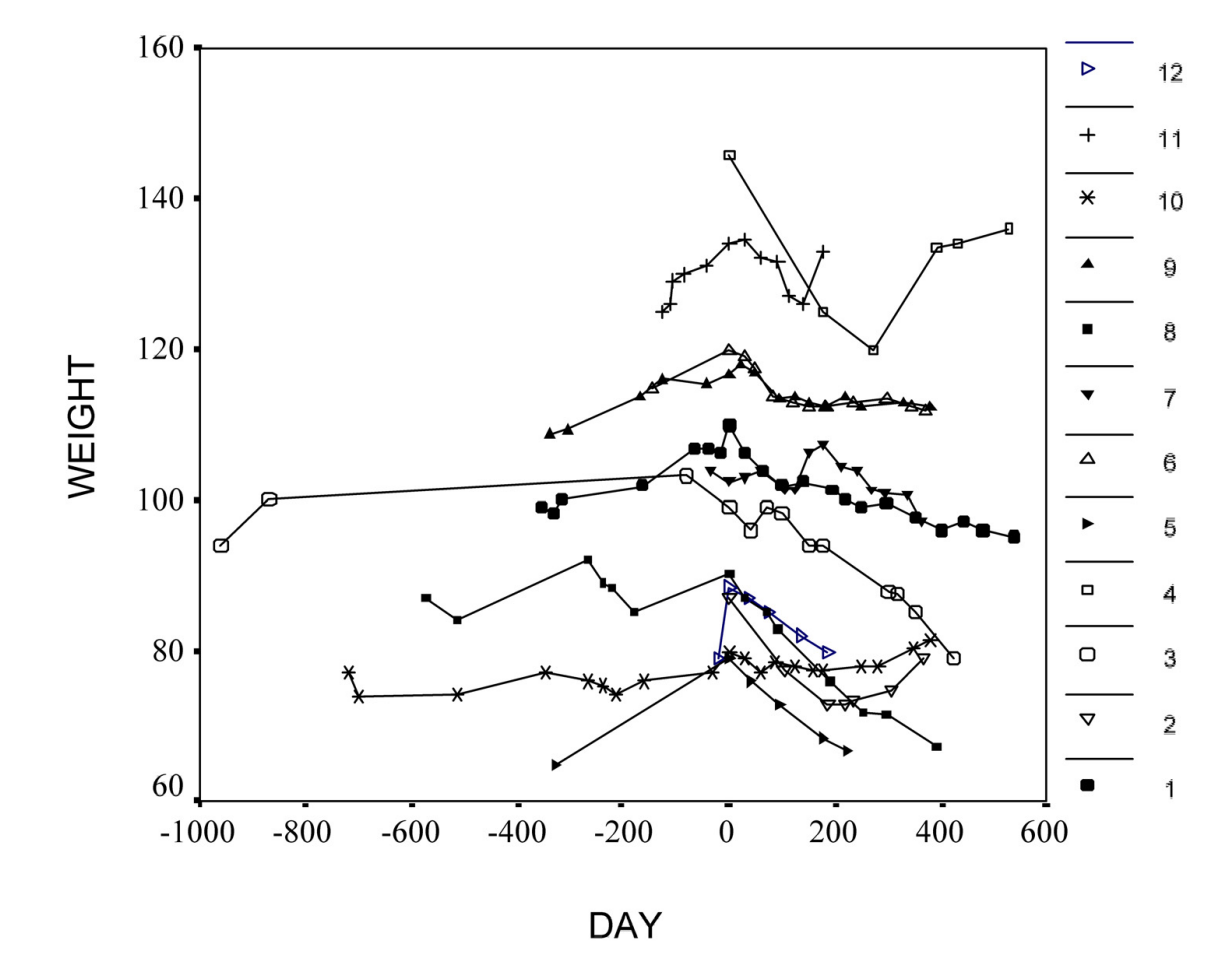
Weight changes in the patients described in the study. The day of treatment is on the x-axis. The day when topiramate was started is day 0 and weight measurements before that point are supplied where available. Each patient's number as referred to in the text is given on the right of the figure.

Patients have received topiramate for between 6 months and one year by the time of writing this report. Six of them are still continuing the treatment. The weight changes are shown in Figure 1 and Table 1. All post-treatment levels and the pre-treatment levels from the previous two years, where available, are shown in Figure 1. At 3 and 6 months, 12 patients lost a mean of 4.96 and 7.75 kg respectively. The nine patients who completed 12 months treatment showed a weight loss of 10.2 and 9.61 kg at the 9^th ^and 12^th ^month respectively. All these changes are significant at p < = 0.005.

### Side effects

One patient (N 5) noticed thinning of her hair and stopped the topiramate after six months. This side effect is known with other mood-stabilizers such as valproate. If true, it is likely to be quite rare, as it has not been mentioned in previous papers, or listed as a side effect. We submitted a report for a suspected adverse drug reaction to the Committee on Safety of Medicines in the UK. One patient (N 10) complained of paraesthesia, sore tongue and bad taste in her mouth and stopped topiramate during an earlier treatment (on Figure 1 this coincides with an earlier short period of weight loss). She restarted it 7 months later (as her weight had increased again by 5.5 kg). This time the medication was titrated more slowly by 25 mg/day every two weeks and she did not experience these side effects. This second start of treatment with topiramate is taken as day 0 for the purposes of this report, as it was of a longer duration and is still on-going. One patient (N 2) complained of paraesthesia, later developed unsteadiness of gait and finally severe Parkinsonism, therefore most of her medications, including topiramate were stopped. This patient was taking a number of psychotropic and cardiovascular drugs (a total of nine!), so we do not know whether topiramate was to blame for her neurological signs, especially as she had been taking this drug for over one year already. She recovered completely around 4 months later and no cause for her condition was identified, despite numerous investigations and consultations. We cannot exclude the possibility that topiramate was at least partially responsible, alone, or in combination with several other drugs, therefore we report this event here. Two patients complained of memory and concentration problems but preferred to continue with the medication despite this. These side effects are also well recognised. One patient did not like the appetite suppressing effect of topiramate and stopped it after only one month, so his data is not analysed in the study.

### Clinical changes

We do not perform regular formal ratings of the mood of our patients. However we regularly record their condition and changes since the previous appointment. Clear improvement of the course of illness was noticed only in one patient (N 3), while eight patients continued to display a similar pattern of illness, as they had done before the treatment. None of them was judged to have deteriorated. The patients in remission remained well. One patient (N 12) whose carbamazepine was changed to topiramate developed a manic episode, therefore we stopped his topiramate after 6 months and asked him to resume carbamazepine.

## Discussion

This is a naturalistic case-series and the absence of a placebo control group could have influenced the results. On the other hand, these patients had already made unsuccessful efforts to lose weight and, as can be seen on Figure 1, their pre-topiramate weight had been either stable, or in most cases had increased steadily during the period of observation. The weight-reducing potential of topiramate was impressive in 10 of the 12 patients and the effect was maintained for the duration of the observation, in most cases for more than one year. These results are in keeping with a large body of evidence supporting the weight-losing potential of topiramate, as presented in the Background. In fact, we could not find a single study that reported a mean weight gain with topiramate. The weight loss in our patients was present already at the third month (mean = 4.96 kg, SD = 3.33, p < 0.001) and continued to increase at least until the 9^th ^month. Only one patient (N 10) had a weight gain or 0.5 kg at the end of the observation which still represented an improvement on her tendency to gain weight in the previous years.

Obesity is a very common problem in developed countries. Its prevalence in the USA is rising and has been estimated at 22.5% among adults aged 20 and over in the year 2001 [38]. Weight gain is even more prevalent in bipolar disorder patients [2]. The increased weight in this population is due to a number of factors, including life-style and diet, but a major cause is the side effect profile of the medications that are prescribed long-term for their illness. If deaths due to suicide and accidents are excluded, there is still a substantially increased mortality in patients with mood disorders, which is due mostly to circulatory disorders [39,40]. Increased weight, high levels of smoking and reduced exercise are likely to be the main factors leading to such an increased mortality. Health professionals cannot just give advice on diet and exercise to overweight patients, because the weight gain is to a large extent caused by the more sedentary life imposed by the illness, combined with the side effects of the majority of drugs they are prescribed. Psychiatrists should have a responsibility in managing obesity in their patients by choosing more appropriate drugs in patients prone to weight gain. This will bring several benefits: 1) It will improve the quality of life of their patients, as weight gain is among the most distressing side effects of psychotropic drugs [41]. 2) A reduction in weight could increase their life-expectancy, as weight loss can reduce mortality [1,11]. 3) An improved weight can increase the adherence to long-term treatment with mood stabilizers.

Several studies, including this one, show that topiramate has a strong potential to induce weight loss which is sustained for at least one year. Despite the side effects reported in this and other studies, topiramate is generally well tolerated. Most of the patients in our series who complained of side effects, still preferred to continue the treatment, as they liked its overall effect. In our experience it is very unusual to have such a high rate of patients who continue to take a new medication. The effect on memory and concentration should however receive closer examination in future studies.

The main unanswered question is whether topiramate has any mood-stabilizing properties, like other anticonvulsants such as carbamazepine, valproate and lamotrigine. Open studies for maintenance treatment in bipolar disorder and in the acute treatment of mania have so far been encouraging (as reviewed in the Background). However, four large unpublished placebo-controlled monotherapy trials failed to confirm the efficacy of topiramate in the treatment of acute mania (cited in [35]), indicating a poor antimanic effect, at least in monotherapy. Our work was not designed to examine any mood-stabilizing properties of topiramate, as we did not use regular mood charts, the period of 12 months is too short to evaluate long-term benefits on the pattern of episodes, and there was no comparison group. In addition, many of our patients were already talking a combination of mood stabilizers without complete treatment effect, having failed to achieve a long-term stability on a variety of treatments that had been tried over the years. In view of that, we did not expect any clear improvements in the mood or course of illness of this patient population. All we can conclude so far is that topiramate does not appear to be superior to other mood stabilizers in the long-term treatment of patients with treatment resistant affective disorders. One of our patients had a manic relapse after we changed his carbamazepine with topiramate (he was also receiving regular lithium prophylaxis). In addition, we would like to report that topiramate was given to two more patients in our clinic who had a BMI of <30 kg/m^2 ^and are therefore not presented as part of this case-series. These two patients had refused to take other mood-stabilizing drugs for fear of weight gain and only agreed to take topiramate as monotherapy, having heard that it will not cause weight gain. Both of them suffered relapses of mania, which necessitated the addition of atypical antipsychotics and admission to hospital. Although these three observations are anecdotal, they strengthen the impression that topiramate monotherapy does not have antimanic effect. Whether topiramate has any effect on the depressive side of bipolar illness is not yet know.

## Conclusion

Topiramate appears a very useful drug for weight reduction in patients with bipolar disorders. The mood stabilizing effect of topiramate is however questionable. The very different results obtained from the controlled monotherapy trials of topiramate in mania and the open add-on trials summarised in the Background indicate that topiramate should not be prescribed as monotherapy in bipolar disorders as it has no acute antimanic effect. In our opinion the current place of topiramate in the treatment of affective disorders is as an add-on treatment for patients who experience clinically significant weight gain, which could either compromise their physical health, or influence them to stop taking established mood stabilizers.

### Recommendations

**Topiramate should not be used in monotherapy **and unless new research shows otherwise, psychiatrists should assume that it has no mood-stabilizing properties.

#### Dose titration

Topiramate dose should be increased gradually and in two-weekly intervals.

#### Cognitive function

Topiramate is known to cause neurocognitive side effects. Psychiatrists should monitor their patients carefully for the emergence of such side effects.

#### Pregnancy

As with most other mood-stabilizers (anticonvulsants and lithium), women who may become pregnant should be warned of the possible consequences of taking such drugs during pregnancy [37].

#### Adequate hydration

Patients should be advised to ensure adequate hydration, especially if they are predisposed to nephrolithiasis [37].

## Competing interests

G. Kirov has received a grant from the Janssen Research Foundation for the collection of families with psychiatric disorders in Bulgaria for genetic studies. The current work is completely independent of that study, we have not informed the company of our work with topiramate, and have not shown them the manuscript. The Mood Disorders Clinic in Cardiff does not receive financial or any other support from Janssen-Cilag (Janssen-Cilag is the producer of topiramate). G. Kirov has received honoraria for lecturing from Eli Lilly and AstraZeneca and grant support from the Wellcome Trust, MRC, London, and Sanofi Synthelabo. J. Tredget's post is funded by AstraZeneca. None of the financial support received by the authors has been in any way related with the work described in this paper.

## Authors' contributions

GK was the psychiatrist in charge for the patients reported in this study. He performed most of the analyses and wrote the draft of the paper.

JT is a Research Nurse who was closely involved in the day-to-day monitoring and supervision of the patients, performed some of the assessments, and organised the running of the clinic, thus contributing significantly towards data acquisition. He revised critically the paper and contributed towards the analysis and discussion.

## Pre-publication history

The pre-publication history for this paper can be accessed here:


